# The Medical Schools of British Hospitals: St. Bartholomew's Medical School

**Published:** 1915-11-13

**Authors:** 


### PATRIOTISM IN MEDICINE.

### THE MEDICAL SCHOOLS OF BRITISH HOSPITALS.

It is hard to estimate whether most glory is reflected on the hospitals connected with medical schools by their former students or by the present members of the staff and schools now serving in the war. On the one hand, the cumulative effect of surveying the activities of past students is immense, and such as can never be effaced from memory. On the other hand, the real test of patriotism may be said to lie in the ready surrender at the national call to arms of most of those who were pillars in the establishment. To gain anything like realisation of what the sacrifices of the medical profession have been and are during this war, it is necessary to contemplate what is being achieved both by the past and the present members of the medical schools. Not many authorities have attempted the stupendous task of collecting and tabulating the names of all their *alumni* at present doing duty in the wide field of war. But in one or two conspicuous instances this task has been carried through with an ability which renders the documents in which the results are embodied invaluable contributions to the war literature of the future. To pore over the figures is to be left speculating on the hopeless slough of misery to which our soldiers must, it would seem, have been exposed had not British practitioners rallied from all quarters of the globe to do them service and share their labours.

### St. Bartholomew's Medical School.

A glance at the *rôle* played by the men of St. Bartholomew's comes as a revelation of the influence exerted by members of this great medical school on the event. The following *précis*, compiled from lists supplied by St. Bartholomew's Hospital, of the varied branches of the service to which this body of St. Bartholomew's men is attached will serve better than any comment to illustrate the work they are accomplishing. All ranks and conditions are here massed together, from the men of world fame who are acting as consultants to the Army, and the Generals directing the various medical services, to the young, newly qualified practitioner, the dresser, and the student.

Précis of Distribution of St. Bartholomew's Men.

(1) 136 Royal Naval Medical Service.
(2) 8 Royal Naval Air Service.
(3) 17 Deputy Directors, Assistant Directors, and Deputy Assistant Directors of the Army Medical Service.
(4) 4 Surgeon-Generals, R.A.M.C.
(5) 28 Colonels and Lieutenant-Colonels, R.A.M.C.
(6) 47 Majors and Captains, R.A.M.C.
(7) 272 Temporary Officers, R.A.M.C.
(8) 19 Reserve of Officers, R.A.M.C.
(9) 48 Expeditionary Force, R.A.M.C.
(10) 67 Medical Officers to Territorial Army, R.A.M.C.
(11) 254 Territorial Force, R.A.M.C.
(12) 35 at Military Hospitals, T.F., R.A.M.C.
(13) 96 at Red Cross Hospitals, etc., R.A.M.C.
(14) 23 Commissioned Officers in Regular Army.
(15) 27 Commissioned Officers in Territorial Army.
(16) 15 in the Ranks.
(17) 73 Officers' Training Corps.
(18) 10 Voluntary Aid Detachments.
(19) 108 Indian Medical Service.

**Note.**—*Those in category* 3, *or some of them, are included in the total of categories* 4, 5, *and* 6; *while some of those in category* 9 *may have been included in previous categories. The same remark may apply to other catgories.*

Among this contingent the deaths have been comparatively few. Lieut.-Col. J. W. Jessop, of the Territorial Force, Major Atal, I.M.S., Major Richards, R.A.M.C., Captain Lukis, Captain R. D. O'Connor, Captain B. M. Hughes, Lieut. G. F. Juckes, and Lieut. Armstrong make up the tale of officers killed on duty. To these must unhappily be added the name of Lieut.-Col. G. A. Edsell, who died after being invalided home. Temporary Lieut. E. A. Wright died of septic poisoning, Captain Scot-Skirving and Lieuts. Jones, Banks, and Macadam died of wounds. Surgeon S. C. Searle, R.N., was lost on H.M.S. *Good Hope.* Fourteen are reported as wounded, one of these from gas poisoning, and six are prisoners of war, two of the latter being wounded in addition, while two are safely interned in Holland.

Awards for Gallantry.

A long list of distinctions gained has gladdened the hearts of those who remained at home, and added lustre to the school. No fewer than thirty-nine St. Bartholomew's men have had the honour of being mentioned in Sir John French's dispatches. Col. Sir Anthony Bowlby, C.M.G., has been promoted to K.C.M.G. The Order of Commander of the Bath has been conferred on Surgeon-General T. M. Corker, Col. S. Westcott, C.M.G., Col. Sir W. P. Herringham, and Col. F. W. C. Jones. Lieut.-Col. C. H. Bowle-Evans and Lieut.-Col. H. S. Thurstan have been promoted to C.M.G. Major Fell has been promoted to Brevet Lieut.-Col.

Captains P. A. Lloyd-Jones and H. T. Wilson have gained the D.S.O., and Lieuts. James, Hill and Priestley have been awarded the Military Cross. The Volunteer Officer Decoration has been conferred on the Hon. Surgeon-General Sir C. Pardy Lukis, K.C.S.I., K.H.S. Surgeon B. A. Playne, R.N., has received the D.S.C. for gallantry in rescuing wounded men under fire near Gaba Tepe.

